# An *ARHGEF10* Deletion Is Highly Associated with a Juvenile-Onset Inherited Polyneuropathy in Leonberger and Saint Bernard Dogs

**DOI:** 10.1371/journal.pgen.1004635

**Published:** 2014-10-02

**Authors:** Kari J. Ekenstedt, Doreen Becker, Katie M. Minor, G. Diane Shelton, Edward E. Patterson, Tim Bley, Anna Oevermann, Thomas Bilzer, Tosso Leeb, Cord Drögemüller, James R. Mickelson

**Affiliations:** 1 Department of Veterinary and Biomedical Sciences, College of Veterinary Medicine, University of Minnesota, St. Paul, Minnesota, United States of America; 2 Institute of Genetics, Vetsuisse Faculty, University of Bern, Bern, Switzerland; 3 Comparative Neuromuscular Laboratory, Department of Pathology, University of California San Diego, La Jolla, California, United States of America; 4 Department of Veterinary Clinical Sciences, College of Veterinary Medicine, University of Minnesota, St. Paul, Minnesota, United States of America; 5 Small Animal Clinic, Neruology, Vetsuisse Faculty, University of Bern, Bern, Switzerland; 6 Neurocenter, Department of Clinical Research and Veterinary Public Health, Vetsuisse Faculty, University of Bern, Bern, Switzerland; 7 Institute of Neuropathology, Heinrich-Heine-University Düsseldorf, Düsseldorf, Germany; Uppsala University, Sweden

## Abstract

An inherited polyneuropathy (PN) observed in Leonberger dogs has clinical similarities to a genetically heterogeneous group of peripheral neuropathies termed Charcot-Marie-Tooth (CMT) disease in humans. The Leonberger disorder is a severe, juvenile-onset, chronic, progressive, and mixed PN, characterized by exercise intolerance, gait abnormalities and muscle atrophy of the pelvic limbs, as well as inspiratory stridor and dyspnea. We mapped a PN locus in Leonbergers to a 250 kb region on canine chromosome 16 (P_raw_ = 1.16×10^−10^, P_genome, corrected_ = 0.006) utilizing a high-density SNP array. Within this interval is the *ARHGEF10* gene, a member of the rho family of GTPases known to be involved in neuronal growth and axonal migration, and implicated in human hypomyelination. *ARHGEF10* sequencing identified a 10 bp deletion in affected dogs that removes four nucleotides from the 3′-end of exon 17 and six nucleotides from the 5′-end of intron 17 (c.1955_1958+6delCACGGTGAGC). This eliminates the 3′-splice junction of exon 17, creates an alternate splice site immediately downstream in which the processed mRNA contains a frame shift, and generates a premature stop codon predicted to truncate approximately 50% of the protein. Homozygosity for the deletion was highly associated with the severe juvenile-onset PN phenotype in both Leonberger and Saint Bernard dogs. The overall clinical picture of PN in these breeds, and the effects of sex and heterozygosity of the *ARHGEF10* deletion, are less clear due to the likely presence of other forms of PN with variable ages of onset and severity of clinical signs. This is the first documented severe polyneuropathy associated with a mutation in *ARHGEF10* in any species.

## Author Summary

Leonberger dogs are a breed originally produced by crossing large-bodied dogs, including Saint Bernards and Newfoundlands. A peripheral neuropathy has been described in Leonbergers that is similar to a group of inherited polyneuropathies known as Charcot-Marie-Tooth disease in humans. We collected a cohort of well-characterized Leonberger polyneuropathy cases and controls, conducted a genome-wide association study, and ultimately identified a highly associated and likely causative mutation in the *AHGEF10* gene. This sequence variant is a 10-bp deletion encompassing a splice site, which forces use of a downstream splice site to create a processed mRNA with a premature stop codon, and represents a loss-of-function mutation. The identical mutation was also found in several polyneuropathy-affected Saint Bernards. When homozygous, this deletion results in the onset of clinical signs before four years of age. *ARHGEF10* has not previously been associated with severe CMT, but comes from a family of genes shown to be involved in neuron morphology. This first-documented severe polyneuropathy associated with an *ARHGEF10* mutation in any species provides an opportunity to gain further insights into the pathobiology of diseases associated with this gene. The *ARHGEF10* mutation does not, however, by itself account for all cases of polyneuropathy in Leonbergers or Saint Bernards.

## Introduction

Charcot-Marie-Tooth (CMT) disease is a heterogeneous mix of hereditary motor and sensory neuropathies in humans, for which mutations in more than thirty genes have already been described (Inherited Peripheral Neuropathies Mutation Database). CMT disease is the most common inherited disorder of the peripheral nervous system in humans, affecting an estimated 8 to 41 per 100,000 globally [1]. Classification of CMT cases has historically been based on clinical and electrophysiological findings, histopathology, and the pattern of inheritance, but is now increasingly based on the underlying genetic defect [2]. Unfortunately, the molecular basis of many rare CMT forms remains unsolved. With no curative therapy for human patients with CMT, suitable animal models for development of therapeutic agents and other treatment modalities are highly desirable [3]. Many of the described muscle and peripheral nerve diseases in dogs are suspected to be inherited [4], and it has been suggested that many canine inherited neuropathies strongly resemble forms of CMT disease [5], [6]. The recent identification of mutations in the *NDRG1* (ENSCAFG00000001141) gene in Greyhounds [7] and Alaskan Malamutes [8] with early-onset polyneuropathy supports this hypothesis.

An inherited polyneuropathy (PN) in Leonberger dogs [9] also demonstrates striking similarities to an intermediate form of CMT disease. Leonbergers are a large-bodied breed, reaching adult weights of 45 to 77 kilograms. PN in this breed is characterized by generalized weakness, hypotonia, and muscle atrophy secondary to denervation, particularly of the pelvic limbs [9]. Affected dogs frequently present with a high-stepping pelvic limb gait (pseudo-hypermetria of the hock) [6], decreased or absent tendon reflexes, and changes associated with degeneration of the recurrent laryngeal nerve, including inspiratory stridor resulting from laryngeal paralysis. The age-of-onset of clinical signs can vary from <1 year up to 11 years of age; however, the juvenile-onset patients typically have a more severe and rapidly progressing course of disease. Peroneal nerve biopsies show decreased myelinated fiber density resulting from axonal degeneration and endoneurial fibrosis indicative of chronic nerve fiber loss. Cranial tibial muscle biopsies demonstrate neurogenic atrophy and fatty replacement of muscle fibers indicative of chronic denervation [9]. There is no effective direct therapy for these pathologies, although some relief can be achieved for laryngeal problems by arytenoid cartilage lateralization (called a “tieback” surgery).

A predominance of affected males led to the initial suspicion that PN in Leonbergers was an X-linked disorder, although autosomal recessive inheritance could not be ruled out [9]. A more recent multi-generation pedigree analysis also concluded an X-linked mode of inheritance, with possible influence by other loci that impact age-of-onset and severity of signs [10]. Nevertheless, numerous affected Leonberger females with juvenile-onset PN, coupled with increasing recognition of a wide range in ages of onset and clinical severity of PN, indicate that the underlying genetic mechanisms responsible for PN in this breed remain to be defined. We therefore performed a genome-wide association study (GWAS) with high-density canine SNP arrays in cohorts of Leonberger PN cases and controls, with the aim of identifying genetic defect(s) that contribute to PN. A loss-of-function *ARHGEF10* (ENSCAFG00000013667) deletion was identified which, although not explanatory for all Leonberger polyneuropathy, is highly-associated with a juvenile-onset form of the disease.

## Results

### Genome-Wide Association Mapping

**Figure 1:**
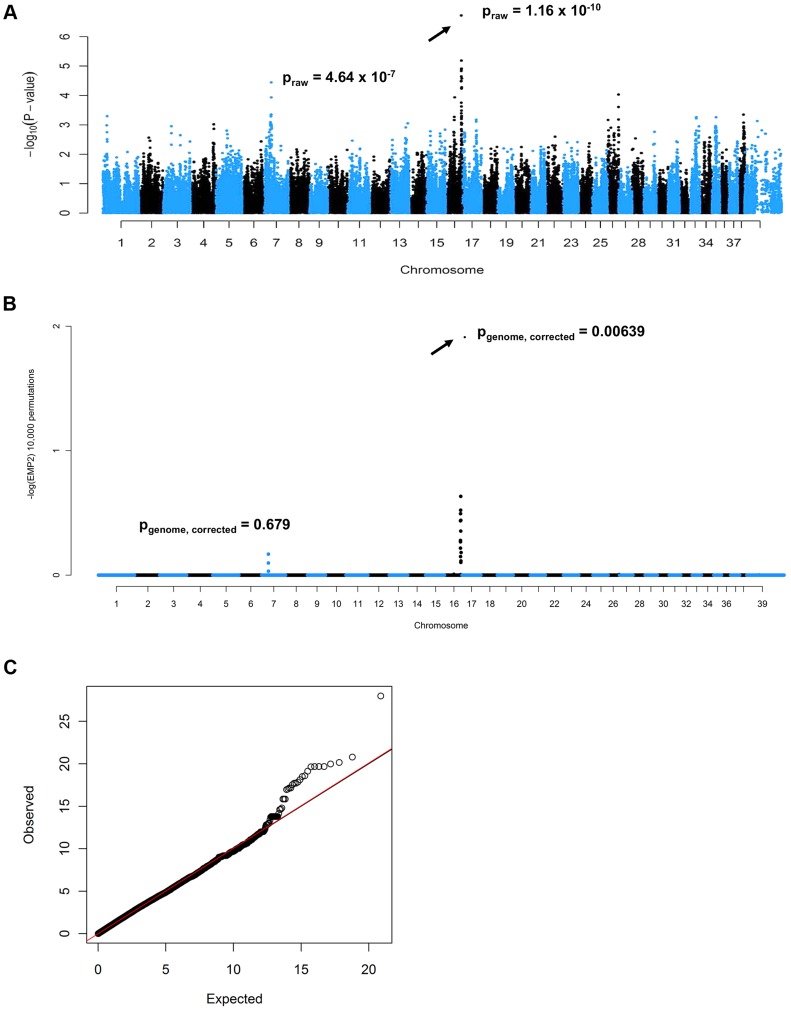
Genome-wide association mapping of Leonberger PN. (A) Manhattan plot of GWAS for PN with 93 (52 cases, 41 controls) Leonbergers. X-axis = chromosome, Y-axis = negative decadic logarithms of permutation-corrected P-values. (B) Manhattan plot of GWAS for PN with 93 Leonbergers. X-axis = chromosome, Y-axis = negative decadic logarithms of permutation-corrected P-values, after correction for population stratification and cryptic relatedness using a mixed model approach and genomic control. (C) The quantile-quantile (QQ) plot corresponding to (B).

A cohort of 52 PN cases and 41 controls was genotyped on Illumina CanineHD BeadChips. After pruning for low genotyping rate, low minor allele frequencies, and failure to meet Hardy-Weinberg equilibrium, 101,284 SNPs remained for the association analysis, which was initially conducted in PLINK [11]. A highly significant CFA16 locus was identified, with the most significant SNP (BICF2G630820235, at bp position 57,375,008, using CanFam2 positions) achieving a raw P-value of 1.16×10^−10^, and a genome-wide P-value of 1.00×10^−4^ after 10,000 permutations (Figure 1A). A potential locus was also observed on CFA7, with the best associated SNP (BICF2P881479, at bp position 25,815,288) achieving a raw P-value of 4.64×10^−7^, and a genome-wide P-value of 0.015 after 10,000 permutations. A genomic inflation factor (lambda) of 1.38 indicated the presence of population stratification and possible cryptic relatedness. This was unsurprising, given that Leonbergers are a breed globally small in numbers, and due to this demographic history, the dogs selected for genotyping on the SNP arrays were unavoidably closely related. We therefore performed an association analysis using the mixed model function implemented in GenABEL [12] that resulted in lambda dropping to 1.004. The same CFA16 and CFA7 SNPs achieved the lowest P-values: 1.90×10^−7^ and 5.79×10^−5^ for CFA16 and CFA7, respectively (Figure 1B). However, with 10,000 permutations of the mixed model test, the CFA16 locus remained significant (P_genome, corrected_ = 0.006), while the CFA7 SNP achieved a p-value of only 0.68 (P_genome, corrected_). The quantile-quantile plot of observed versus expected P-values of this mixed model permuted analysis also supports the effectiveness of the correction for population structure and the significance of the CFA16 locus (Figure 1C).

### Identification and Characterization of an *ARHGEF10* Mutation

**Figure 2:**
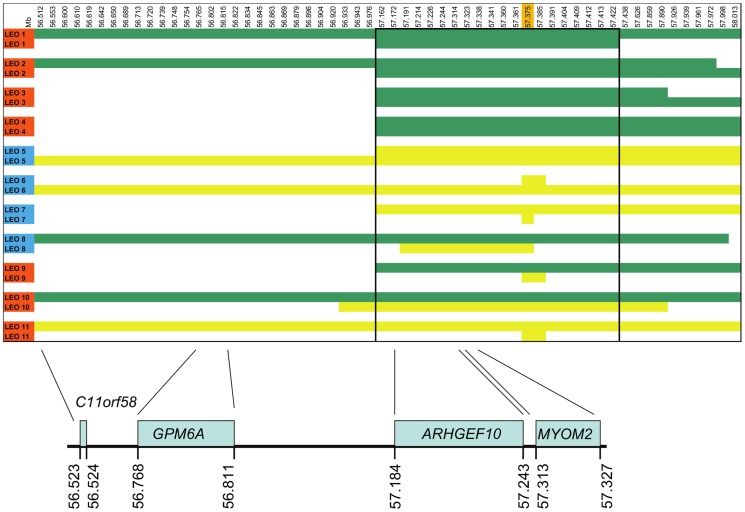
SNP haplotypes and genes from the GWAS identified CFA16 region associated with Leonberger PN. A representative sample of the observed CFA16 haplotypes is demonstrated using eleven Leonbergers, with both haplotypes shown for each dog as green or yellow bars. LEO 1-4 and LEO 9 - 11 are cases (indicated in red), while LEO 5-8 are controls (indicated in blue). The most significantly associated SNP (BICF2G630820235) is highlighted in orange. Green bars represent the most significantly associated haplotype, which, in some cases extended the entire 1.5 Mb region. However, a minimum associated haplotype of approximately 250 kb (enclosed in the dark outlined box) was observed. An alternate haplotype, demonstrated by the yellow bars, was present in many of the controls and some of the cases. Some PN cases (LEO 9-11) are either heterozygous or devoid of the “green” case-associated haplotype and a phenotypically normal dog (LEO 8) is heterozygous for the associated haplotype. CFA16 (Mb, CanFam2) ENSEMBL-annotated genes in the region are shown at the bottom of the figure.

**Figure 3:**
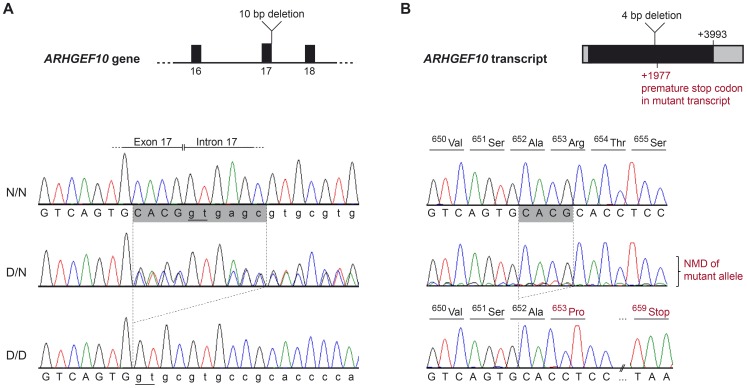
*ARHGEF10* mutation analysis. (A) Genomic DNA. Electropherograms of the *ARHGEF10* c.1955_1958+6delCACGGTGAGC mutation. Representative sequence traces of PCR products amplified from genomic DNA of 3 dogs with the different genotypes are shown. (B) Transcript. Electropherograms of RT-PCR using primers in exon 17 and 19. Representative sequence traces of RT-PCR products amplified from cDNA of 3 dogs with the different genotypes are shown. Note the underrepresentation of the mutant transcript in the heterozygous dog, which is probably due to nonsense mediated decay (NMD). Both the normal and the new splice site created by the deletion are underlined (Panel A); the cryptic splice site in the deletion allele creates a cDNA missing only four base pairs, which creates a frame shift and predicts a premature stop codon within seven amino acid residues (Panel B).

Shared homozygosity in many PN-affected dogs, and absence of homozygosity in controls, identified a 250 kb minimum haplotype at the CFA16 locus. This interval contains only two genes: rho guanine nucleotide exchange factor 10 (*ARHGEF10*) and myomesin 2 (*MYOM2*, ENSCAFG00000024660) (Figure 2). *ARHGEF10* was pursued as a positional and functional candidate gene due to its previous implication in peripheral nerve development and human peripheral hypomyelination [13]. Similar to humans, the canine *ARHGEF10* gene has 29 coding exons, with two alternative transcripts that either include or exclude exon 9, encoding a longer and shorter form of the protein. We first sequenced all coding exons, along with flanking intron boundaries, in four juvenile-onset severely-affected dogs, and four control dogs. This analysis revealed 98 sequence variants in comparison to the canine reference genome sequence (Table S1). Two SNPs predicted an amino acid change that was observed in both cases and controls, and the other exonic variants were synonymous. Two of the four cases used for sequencing were homozygous for the disease-associated CFA16 haplotype, and both of these dogs possessed a private 10 bp deletion (c.1955_1958+6delCACGGTGAGC). This deletion removes four nucleotides from the 3′-end of exon 17 (nts 1955 to 1958) and six nucleotides from the 5′-end of intron 17 (nts 1958+1 to +6) (Figure 3A). A dog that is heterozygous for the CFA16 haplotype (*D/N*) was subsequently sequenced and the results were in accord with heterozygosity for the 10 bp deletion (Figure 3A).

The effect of the deletion on the *ARHGEF10* transcript was investigated with cDNA prepared from the nervous tissue of an early-onset Leonberger PN case homozygous for the CFA16 haplotype (*D/D*), a CFA16 haplotype heterozygote (*D/N*) Leonberger with no polyneuropathy, and one control dog (*N/N*). Sequencing of an RT-PCR product containing the exon 17- exon 18 junction showed that mRNA produced in the PN-affected Leonberger, but not the control, lacked 4 bp from the 3′-end of exon 17, while containing the entirety of exon 18 (Figure 3B). This is consistent with the utilization of an alternate GT splice site immediately downstream from the normal splice site, which is deleted. The resultant mutant transcript contains a frame-shift which predicts a truncation of 50.5% of the amino acid sequence from the long form of the protein (52% of the short form). This predicts deletion of a WD40-like domain and two transmembrane segments. We detected only wild type sequence from the cDNA of the heterozygote, most likely due to poor amplification of the mutant sequence relative to wild type that would result from nonsense-mediated decay.

All 93 Leonbergers from the original GWAS cohort were then genotyped for the *ARHGEF10* deletion (Table 1). Eighteen of the 52 (35%) cases were homozygous for the deletion (*D/D*), 14 (27%) were heterozygous (*D/N*), and 20 (38%) were homozygous normal (*N/N*). The 18 *D/D* dogs represented both sexes and developed clinical signs at or before the age of three years; most did so before the age of two. Three *D/N* dogs and eight *N/N* dogs also developed PN signs at ≤3 years of age. None of the original 41 controls were *D/D*, four out of 41 (10%) were heterozygous *D/N*, and 37 (90%) were *N/N*.

**Table 1:** Genotype, sex, and age-of-onset distribution for well-phenotyped case and control Leonbergers.

					Cases												Controls					
CFA16	Genotype P-value (without splitting sexes or age-of-onset)	Allele P-value (without splitting sexes or age-of-onset)	Total Cases	Total Controls	Males ≤3	Females ≤3	Males >3	Females >3	Males ≤3	Females ≤3	Males >3	Females >3	Males ≤3	Females ≤3	Males >3	Females >3	Males	Females	Males	Females	Males	Females
*ARHGEF10* deletion					*D/D*	*D/D*	*D/D*	*D/D*	*D/N*	*D/N*	*D/N*	*D/N*	*N/N*	*N/N*	*N/N*	*N/N*	*D/D*	*D/D*	*D/N*	*D/N*	*N/N*	*N/N*
Original GWAS	9.60E-07	1.16E-10	52	41	**14**	**4**	**0**	**0**	**3**	**0**	**9**	**2**	**7**	**1**	**7**	**5**	**0**	**0**	**0**	**4**	**12**	**25**
Validation Group	9.57E-06	5.98E-07	154	160	**16**	**2**	**0**	**1**	**4**	**1**	**13**	**13**	**22**	**7**	**51**	**24**	**0**	**0**	**12**	**14**	**59**	**75**
All cases/controls meeting inclusion criteria	5.01E-11	2.75E-15	206	201	**30**	**6**	**0**	**1**	**7**	**1**	**22**	**15**	**29**	**8**	**58**	**29**	**0**	**0**	**12**	**18**	**71**	**100**

					Cases												Controls					
CFA7	Genotype P-value (without splitting sexes or age-of-onset)	Allele P-value (without splitting sexes or age-of-onset)	Total Cases	Total Controls	Males ≤3	Females ≤3	Males >3	Females >3	Males ≤3	Females ≤3	Males >3	Females >3	Males ≤3	Females ≤3	Males >3	Females >3	Males	Females	Males	Females	Males	Females
*TIGRP2P93473_rs9189862*					*G/G*	*G/G*	*G/G*	*G/G*	*G/A*	*G/A*	*G/A*	*G/A*	*A/A*	*A/A*	*A/A*	*A/A*	*G/G*	*G/G*	*G/A*	*G/A*	*A/A*	*A/A*
Original GWAS	2.85E-05	2.90E-06	52	41	**3**	**0**	**3**	**2**	**12**	**4**	**6**	**4**	**9**	**1**	**7**	**1**	**0**	**0**	**2**	**6**	**10**	**23**
All cases/controls meeting inclusion criteria	7.80E-02	0.043	193	174	**4**	**1**	**11**	**6**	**28**	**9**	**24**	**14**	**25**	**5**	**41**	**25**	**4**	**5**	**27**	**39**	**42**	**57**

Top: CFA16 *ARHGEF10* deletion genotyping of the original GWAS cohort, the larger, independent validation group, and all dogs combined. *D/D* = homozygous for the *ARHGEF10* deletion. *D/N* = heterozygous for the *ARHGEF10* deletion. *N/N* = homozygous normal. Each genotype group is further broken into sex and age-of-onset of clinical signs (either before or during the dog's third year, or after the dog's third year). Chi-square test of homogeneity p-values are reported for both genotypic and allelic frequencies (although these were calculated without using the sex or age-of-onset splits). Bottom: CFA7 TIGRP2P93473_rs9189862 SNP (located at Mb position 25,888,992) genotyping of the original GWAS cohort, and a combined cohort of all cases and controls meeting the inclusion criteria (GWAS dogs are included here). CFA: *Canis lupus familiaris* chromosome; GWAS: genome-wide association study.

### Effects of Genotype, Sex, and Age on the PN Phenotype

We next genotyped a larger and independent population of case (n = 154) and control (n = 160) Leonbergers (“Validation Group”) (Table 1), where the association of the *ARHGEF10* mutation with PN was confirmed (genotypic and allelic P-values of 9.57×10^−6^ and 5.98×10^−7^, respectively). When combined, the GWAS and validation populations comprised 206 cases and 201 controls, which increased the significance of the genotypic and allelic frequency differences (P-values of 5.01×10^−11^ and 2.75×10^−15^, respectively).

**Figure 4:**
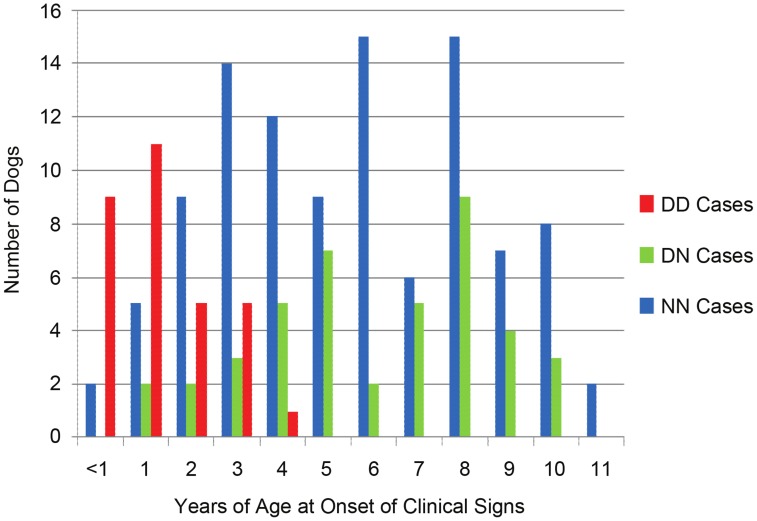
Age-of-onset based on *ARHGEF10* genotype. Age-of-onset of clinical signs for all well-phenotyped cases (where age-of-onset was reported) is shown for all cases from the original GWAS and the independent, validation group, by *ARHGEF10* genotype. Not all 206 dogs are shown due to the fact that, while we sometimes knew the dog had clinical signs by a certain age, the exact onset of those signs before that age was unknown.

Overall, 44.4% (36/81) of young age-of-onset (≤3 years) cases had the *D/D ARHGEF10* genotype (Table 1, Figure 4). Forty-one of the 81 early onset cases had undergone a nerve biopsy that showed pathological changes consistent with PN, and 21 of these (51.2%) were homozygous for the *ARHGEF10* deletion. In total, 90 of the 206 cases had undergone peripheral nerve biopsy to confirm pathological changes consistent with PN, with 21 of them (23.3%) being homozygous for the *ARHGEF10* deletion.

In the combined population, 30 *D/D* cases are male and seven *D/D* cases are female. Affected males outnumber affected females in nearly every category of genotype and age-of-onset (Table 1). When the data is normalized to the number of samples from each sex, 65% of the submitted males were affected and 34% of the submitted females were affected.

The effects of heterozygosity for the *ARHGEF10* deletion were also ascertained in the combined population. Sixty percent (45 of 75) of all *D/N* dogs were PN cases and 40% (30 of 75) were controls (Table 1). *D/N* dogs accounted for 21.8% of all PN cases (45 of 206) and 14.9% of all controls (30 of 201), and represent only a slightly significant difference in “D” allele frequency between cases and controls (P-value = 0.009). The average age-of-onset of clinical signs for cases in the combined population was 1.73, 6.14 and 5.59 years for *D/D*, *D/N* and *N/N* dogs, respectively. Thus heterozygous or homozygous normal PN cases developed clinical signs at a similar later stage in life (Figure 4). Based on our assessment of medical records, PN cases that were heterozygous for the *ARHGEF10* deletion were less likely to develop severe disease than were *D/D* dogs.

A global group of 4,074 Leonbergers, primarily originating from North America and Europe, have been submitted to our laboratories since the discovery of the *ARHGEF10* mutation. In this non-random sample, 1.3% of these dogs were homozygous for the *ARHGEF10* mutation, 15.4% were heterozygous, and the “*D*” allele frequency was 9.0%. There was no significant difference between numbers of *D/D* males (34/54) and *D/D* females (20/54) (P-value = 0.077).

### *ARHGEF10* Deletion in Saint Bernards and Other Breeds

The Leonberger breed resulted from crossing several breeds, including the Saint Bernard, Newfoundland, and Great Pyrenees, making it possible that the *ARHGEF10* mutant allele could be present in these breeds as well. To date we have identified the identical deletion mutation in a homozygous state in four (2 males, 2 females) Saint Bernard dogs with early-onset PN. Of these four dogs, three were confirmed as PN-affected with a nerve biopsy, and all four showed similar clinical signs to those seen in *D/D* Leonbergers, including laryngeal paralysis, pelvic limb deficits and flaccid paraparesis, and severe neurogenic muscle atrophy. All four were deceased by the age of two years as a result of their disease. A random sample of 383 Saint Bernards found 1.8% of them to be heterozygous and 98.2% of them to be homozygous normal. (Note that the four D/D dogs are not included in the random sample, as they were acquired via diagnostic sample submission after showing clinical signs).

Other related breeds, and breeds with clinical PN, have also been genotyped for the *ARHGEF10* deletion and all were *N/N* (Table S2). This includes Newfoundlands (n = 359, three with PN diagnosed by nerve biopsies), Greater Swiss Mountain Dogs (n = 48), Bernese Mountain Dogs (n = 2, both with PN diagnosed by nerve biopsies), Great Pyrenees (n = 7, two with PN diagnosed by nerve biopsies), Great Danes (n = 4, all with PN diagnosed by nerve biopsies), and a single Golden Retriever (also with PN diagnosed by nerve biopsy).

### Follow-up of CFA7

We genotyped one of the most significantly associated CFA7 SNPs (TIGRP2P93473_rs9189862) from the initial GWAS in a large Leonberger population of 193 cases and 174 controls (Table 1). This resulted in a large decrease of both genotypic and allelic significance for this SNP, indicating that the weaker initial association did not maintain significance.

## Discussion

There are only two early-onset peripheral neuropathies with a known genetic basis in dogs; both being autosomal recessive conditions due to mutations in the *NDRG1* gene [7], [8]. PN in the Leonberger was initially considered to be a single, possibly X-linked, disorder [9], [10]. However, we now understand that Leonberger dogs affected with PN can display a broad range of age-of-onset, so it is reasonable to conclude that PN in this breed, like CMT disease in humans, may not be a single disease with one underlying genetic mutation. Nevertheless, we were able to utilize a GWAS strategy to identify a highly significantly associated locus on CFA16. We found a biologically noteworthy positional candidate gene, *ARHGEF10*, that harbored a 10 base deletion affecting the coding sequence. We further confirmed the association of this mutation with juvenile-onset PN in a large, independent validation group of Leonbergers, characterized the effects of genotype, age and sex on the mutation's association with PN, and detected it in cases of early-onset PN in Saint Bernard dogs. *ARHGEF10* belongs to a large family of Rho guanine nucleotide exchange factors (GEFs), which are activators of Rho-GTPases, molecular switches that participate in the regulation of a large number of signal transduction pathways, some of which influence cell polarity, specifically neuron morphology, including axon, dendrite, and spine growth, and axon guidance [14]. RhoGTPases are also specifically involved in directing the migration of Schwann cell precursors along outgrowing axons [15]. As *ARHGEF10* is expressed in multiple tissues, with relatively higher expression in the spinal cord and dorsal root ganglion [13] (and USCS Genome Browser), we hypothesize that the loss of *ARHGEF10* leads to the loss of proper signaling for axon ensheathment and myelination in Leonberger PN.

Autosomal recessive mutations in the rho GTPase GEF frabin/FGD4 gene have been previously implicated in demyelination of peripheral nerves in cases of CMT type 4H [16], [17], and an autosomal dominant point mutation predicting a missense mutation in exon 3 of *ARHGEF10* has been identified in a human family experiencing non-clinical slowed nerve-conduction velocities [13]. A nerve biopsy of the human proband in this family revealed numerous thinly myelinated axons, without any gross signs of demyelination or axonal degeneration and regeneration, and none of the affected family members in the study experienced progression of symptoms. Subsequent work examining this human *ARHGEF10* mutation concluded that the relatively mild phenotype is due to activation of GEF activity [18]. Conversely, Leonberger PN cases in the present study have decreased nerve fiber density, and chronic nerve fiber loss, resulting from axonal degeneration with progressive clinical signs of weakness and muscle atrophy. Given that the *ARHGEF10* deletion in Leonbergers truncates approximately 50% of the protein, and the deleted portions contained the WD40-like domain and both transmembrane segments [19], it is reasonable to suggest that the severe and progressive clinical and pathological phenotype observed in Leonbergers homozygous for the mutation is due to the loss-of-function of this GEF's activity. This is the first report of an *ARHGEF10* mutation in any species resulting in a severe juvenile-onset PN.

Our data suggests that LPN1 (PN due solely to the *ARHGEF10* mutation) is most likely inherited in an autosomal recessive manner and explains approximately one-fifth of all PN cases in Leonbergers. There is a slight excess of D/N heterozygote cases (compared to controls) (Table 1); this could be due to: 1) interaction with another locus, 2) the possibility that the deletion is actually semidominant, in which case, all homozygous D/D dogs become affected early in life, while heterozygous D/N dogs have an elevated risk of developing less severe disease later in life compared to truly normal N/N dogs, 3) that there are one or several rare additional undetected *ARHGEF10* variants, or 4) sampling bias in the case population. A semidominant mode of inheritance seems doubtful, due to the fact that we have observed many D/N dogs without clinical signs of PN. Although we favor the recessive model, we cannot, as yet, entirely rule out any of these hypotheses. It has been recognized in several studies [20]–[24] that laryngeal paralysis is a strong indicator of the presence of an underlying ‘silent’ polyneuropathy that may later progress; this is likely the scenario with many of our later-onset Leonberger cases, whose clinical presentation is different than dogs with juvenile-onset. Therefore, we suggest that additional cases of PN could be due to other unidentified PN-causing mutation(s), and may be influenced by age, sex, environmental or other genetic factors.

A sex bias in submitted PN cases toward males is evident, as 70% of all cases were male, and 65% of all males in the study were affected, as compared to 37% of all females. Nevertheless, male and female homozygotes for the *ARHGEF10* deletion exhibit the same classic clinical signs of peripheral PN, and in the global population of >4,000 genotyped Leonbergers there was no statistical difference in sex distribution of *D/D* cases. The identification of an autosomal PN-causing *ARHGEF10* mutation, and no indication of association of PN to SNPs on the X chromosome in our GWAS, contradicts the previously assumed X-linked inheritance [9], [10]. Hultin Jaderlund et al. argue from a limited pedigree analysis that inherited PN in Leonbergers is likely to be a single disease with an X-linked mode of inheritance, which is genetically different in Europe and the Americas, and descended from a Leonberger female, born in 1943. However, our study, using to the extent possible the gold standard of peripheral nerve biopsy to confirm a diagnosis of PN, combined with mutation identification and analysis on a large, world-wide sample cohort, indicates that: 1) Leonberger PN represents at least two, if not more, distinct inherited diseases, 2) the first identified PN mutation is autosomal, and 3) it is found worldwide.

A possible explanation for the higher number of males affected is that males develop more severe clinical signs at an earlier age because they are typically larger than females, with correspondingly longer peripheral nerves (those typically more susceptible and affected first in peripheral neuropathies [25]). An alternative explanation involves the effects of neuroactive endogenous steroids, such as progesterone, testosterone, and their derivatives on the peripheral nervous system. These steroids originating from both peripheral glands and the nervous system [26] influence neurodegenerative processes as well as signaling pathways involved in neuronal cell death [27]. Neuroactive steroid levels in a rat model of CMT type 1A are sexually dimorphic [28], and a recent study of rat peripheral nerve cell cultures demonstrated a sex difference in the promotion of Schwann cell proliferation by neuroactive steroids [29]. Finally, it has been suggested that sex differences in DNA methylation patterns may be exerted on the developing nervous system by steroid hormones [26]. It is entirely possible, then, that the overall sex-difference in Leonberger peripheral nerve pathology is a result of sex-specific presence and ability to respond to neuroactive steroids and/or epigenetic changes they introduce.

The initially detected suggestive association on CFA7 was a possibility for a second inherited PN locus segregating in the Leonberger breed; however, it was not confirmed in a larger population. Although we might have missed identifying a non-coding regulatory mutation within the associated haplotype, it seems more likely that by random chance the controls selected for the GWAS population had fewer copies of a CFA7 haplotype that is actually quite common in the breed, which in turn misrepresented the allele frequencies and led to the appearance of significant association. While we cannot rule out that this CFA7 locus is involved with a subset of PN cases in Leonbergers, it is likely that a far more powerfully-designed GWAS will be necessary to identify additional PN loci in this breed.

Besides apparent cases of PN caused by different genetic mutations, it is possible that some of the non-LPN1 “cases” in our sample collection are not truly PN and might represent misdiagnosed phenocopies. A gold standard for diagnosing PN would include measurement of motor and sensory nerve conduction velocities (for demyelination), amplitude of the compound muscle action potential (for axonal loss), histopathology of peripheral nerve biopsies and an examination conducted by a board-certified veterinary neurologist. However, since many dogs had not undergone sufficient diagnostic testing, we were unable to use these stringent criteria. Diseases such as hip dysplasia, a torn cruciate ligament, or undiagnosed osteosarcoma, for example, can induce gait abnormalities that mimic those seen in PN. Without a complete assessment, owners, and possibly veterinarians, can easily mistake these diseases, at least in the early stages, with PN. PN phenocopies can also result from primary hypothyroidism, which has been associated with peripheral neuropathy in dogs and can create very similar changes to the nerve appearance on biopsy [30]. Hypothyroidism typically affects middle to older-aged dogs, and especially medium to large-sized dogs [31], [32]. Therefore an older-onset hypothyroid dog could be mistaken for an older-onset PN case. Lastly, a presumptively inherited leukoencephalomyelopathy (LEMP) has been reported in Leonberger dogs [33]. While LEMP is a disease of the central nervous system and no lesions are observed in the peripheral nerves, clinical signs such as ataxia and hypermetria are neurologic in origin, and could be mistaken as inherited peripheral PN. Despite these phenotyping challenges, strict case definitions emphasizing the diagnosis of PN from axonal degeneration and chronic nerve fiber loss observed in a peroneal nerve biopsy allowed us to assemble a strong case population for the initial GWAS.

The Leonberger breed was created in the mid-1800s by a citizen of Leonberg, Germany, who ostensibly crossed a Landseer Newfoundland female with a St. Bernard male. Other breeds, such as the Pyrenean Mountain dog were likely also introduced [6]. The Leonberger breed experienced severe bottlenecks during and after both World Wars, and it is difficult to ascertain when Saint Bernards ceased to be interbred with Leonbergers. Clinically and histopathologically confirmed PN, similar to that of the Leonberger dogs, occurs in the Saint Bernard, Newfoundland, and Great Pyrenees (Shelton, unpublished observation), but of these three, to date the *ARHGEF10* deletion has only been identified in the Saint Bernard. Though it is possible that the *ARHGEF10* deletion mutation pre-dates the formation of the Leonberger breed, and was introduced via the Saint Bernard, it is also possible that the mutation is newer, and was introduced through more recent cross-breeding. We believe it is less likely that the mutation arose in the Leonberger breed and was subsequently introduced to Saint Bernards, given the Saint Bernard's longer pedigreed breed establishment. The low sample numbers tested to date in other breeds does not yet effectively rule-out the presence of this deletion in any other breed of dog.

In conclusion, we have identified a 10 bp deletion in the canine *ARHGEF10* gene as the most likely causative mutation for a juvenile form of PN, now termed LPN1. This mutation explains 20% of all PN-affected Leonbergers and occurs rarely in PN-affected Saint Bernards. Dog breeders can now select against the defective *ARHGEF10* allele by genotyping breeding animals and using targeted mating to achieve a significant reduction of PN incidence; specific breeding recommendations and test result interpretations from our laboratories are included with this manuscript as Supporting Information S1. This work suggests a critical function of *ARHGEF10* in normal development and/or maintenance of peripheral nerves and in so doing we have also defined an excellent animal model for human CMT.

## Materials and Methods

### Sample Collection and Ethics Statement

This study was performed using protocols approved by the Institutional Animal Care and Use Committees (IACUC) of the University of Minnesota (UM), the University of Bern (UB), and the University of California San Diego (UCSD). Written consent was obtained from all dogs' owners. Leonberger samples were obtained primarily via elective owner submission or collected at breed club shows. Medical records, pedigrees, and a blood sample or cheek swab for DNA extraction were requested from each Leonberger dog submitted to the UM and UB. Following identification of the *ARHGEF10* mutation, samples of many more Leonberger dogs were submitted for genotyping. Many of these dogs do not have complete medical information and were used only for a population study. Other dogs represented breeds closely related to the Leonberger (including the Saint Bernard), or had developed signs of clinical polyneuropathy and/or had a nerve biopsy showing pathological changes consistent with PN. Samples submitted to UCSD additionally included nerve and/or muscle biopsies for diagnostic purposes. A frozen Leonberger nerve tissue sample from a PN case, shipped originally to UCSD for diagnostic biopsy purposes, as well as frozen spinal cord tissue from a Leonberger and frozen brain tissue of a Cavalier King Charles Spaniel, both unaffected with polyneuropathy and obtained for purposes of other studies, were used for RNA isolation and cDNA sequencing. Additional DNA samples used to examine frequency of the *ARHGEF10* deletion in breed populations (Newfoundlands, Saint Bernards, and Greater Swiss Mountain Dogs) were collected for other studies and shared by collaborators (see acknowledgement section).

### Case and Control Definitions for 93 Leonberger Dogs Utilized in the GWAS

The clinical characterization of LPN, including morphometric, electrophysiological, biopsy and examination findings, has been described elsewhere [9]. As sample acquisition continued after publication of the original study, it became clear that a large spectrum of clinical signs and substantial variety in the available diagnostic information exists among cases. Therefore, the following criteria were established in order to select the best possible cases and controls for SNP array genotyping:

#### Cases (n = 52)

The dog's age of onset of clinical signs was known. A peripheral nerve biopsy, processed and examined in the Comparative Neuromuscular Laboratory of the author (GDS) or another veterinary neurohistopathologist using proper plastic embedding techniques, was positive for the typical Leonberger polyneuropathy. Clinical signs reported by the owner and/or veterinarian must have included at least one gait abnormality and at least one laryngeal abnormality. Accepted gait abnormalities included: a characteristic high-stepping pelvic limb gait (pseudo-hypermetria of the hock), inability to bear weight, and stumbling. Gait abnormalities were required to be fairly symmetrical. Some degree of atrophy of the pelvic musculature may also have been exhibited. Accepted laryngeal abnormalities included: a change in the bark quality (tone, pitch, etc.), respiratory stridor, difficulty breathing and/or swallowing, laryngeal paresis or laryngeal paralysis (as observed by endoscopic examination) and laryngeal tieback surgery. Other diseases that may mimic any of these signs, such as neoplasias, paraneoplastic syndromes, hypothyroidism, diabetes mellitus, etc., were ruled out to the extent possible. Thirty-six cases met these qualifications. Of the remaining sixteen cases used in the GWAS, a subset (n = 8) met the above qualifications, but only exhibited either a gait abnormality or a laryngeal abnormality, but not both. Another subset (n = 2) did not have a nerve biopsy available, but had undergone nerve conduction velocity (NCV) testing, revealing abnormal (decreased or absent) NCVs. These dogs also had a gait abnormality and a laryngeal abnormality. A final subset (n = 6) had both a gait abnormality and a laryngeal abnormality, but no nerve biopsy and no NCV testing.

These 52 cases included ten of the cases described previously [9]. Twenty-nine (24 male, 5 female) of the cases developed clinical signs of polyneuropathy before or at three years of age, while the other 23 (16 male, 7 female) cases developed signs at an age older than three years. Forty of the 52 cases (77%) were male and 12 of the cases (23%) were female.

#### Controls (n = 41)

The dog must have been at least eight years of age at the time it was genotyped or at the time of death, with no laryngeal abnormalities and no gait abnormalities (as described above) reported. For 14 of the controls, post-mortem peripheral nerve biopsies were processed and examined at UCSD or by another veterinary neurohistopathologist, using proper plastic embedding techniques, and showed no evidence of nerve fiber loss beyond that which is normal for age-related changes. A subset (n = 4) of the controls had all of the above criteria, but were seven years old at the time genotyped or at the time of death. A final subset (n = 23) were over the age of eight and were clear of all clinical signs, but had not been biopsied post-mortem.

### Phenotype Classification of Additional Leonberger Dogs

#### Validation group

To better assess the relationships between genotype and phenotype, as well as sex and age-of-onset of clinical signs, and in order to validate the association between the *ARHGEF10* deletion and PN-affected status, we examined an independent set of Leonberger samples (n = 314). Control dogs (n = 160) were restricted to those aged eight years or older with no laryngeal or gait abnormalities. Cases (n = 154) included possible PN clinical signs, and: either having a nerve biopsy with pathological changes consistent with PN (n = 60 of 154), abnormal NCV test results (n = 2 of 154), a diagnosis for laryngeal paralysis (with or without tieback surgery) and a gait abnormality (n = 43 of 154), or having a gait and breathing abnormality together (n = 49 of 154). Medical information was provided by the owners and veterinarians as above.

#### Global group

To estimate the allele frequencies in a world-wide population of Leonbergers, we utilized the combined sample set that has been received by UM and UB in response to availability of an LPN1 genotyping test (n = 4,074). This represents a sample pool originating primarily from the North American and European continents, as well as Australia/New Zealand. Very few samples have been tested which originated from Asia and South America. The dogs used in the GWAS and the validation group above are pooled in this global total.

### DNA and RNA Extraction

Genomic DNA was isolated from blood or tissue using the Gentra PureGene blood kit (Qiagen, Germantown, USA) according to standard protocols; for tissue, the modified protocol for DNA purification from paraffin-embedded tissues from the same kit was used. Total RNA was isolated using TRIzol (Life Technologies, Grand Island, NY, USA), according to manufacturer's instructions.

### Genome-Wide Association Mapping and Haplotype Analysis

Initially, genomic DNA from 52 cases and 41 controls was genotyped on the Illumina CanineHD BeadChip (Illumina, San Diego, CA, USA) that contains 173,662 SNP markers. SNP genotype data was analyzed in PLINK [11], and subjected to standard quality control, where SNPs were excluded for poor genotyping rates (<90%), low minor allele frequencies (<0.05), or deviation from Hardy Weinberg equilibrium in controls (p<0.001), and dogs were excluded for low genotyping success (<90%). The data were subjected to chi-square tests of association, and 10,000 phenotype label-swapping permutations were utilized to determine genome-wide significance. Population stratification, resulting from close familial relationships, was confirmed by the genomic inflation factor calculated during the chi-square association test. Therefore, we applied a mixed model approach utilizing the GenABEL package [12], with 100,000 permutations, to correct for this population stratification and any cryptic relatedness. Haplotypes around significantly associated loci were constructed using PHASE [34], [35]. All bp positions used are reported from the May 2005 (Broad/CanFam2) build of the canine genome, in order to be synchronized with the SNP arrays.

### Mutation Analysis of Canine *ARHGEF10*

Primers for the amplification of the coding region of *ARHGEF10* were designed with the software Primer 3 after masking repetitive sequences with RepeatMasker. We amplified PCR products (conditions available upon request) using MJ Research PTC-100 thermal cyclers (MJ Research, Inc., Watertown, MA, USA) covering exons and flanking intronic regions using AmpliTaqGold360Mastermix (Life Technologies, Grand Island, NY, USA). Re-sequencing of the PCR products was performed after rAPid alkaline phosphatase (Roche Diagnostics Corporation, Indianapolis, IN, USA) and exonuclease I (New England Biolabs (NEB), Ipswich, MA, USA) treatment using both PCR primers with the ABI BigDye Terminator Sequencing Kit 3.1 (Life Technologies, Grand Island, NY, USA) on an ABI 3730 genetic analyzer. We analyzed the sequence data with Sequencher 5.1 (GeneCodes, Ann Arbor, MI, USA).

We used two assays to genotype additional dogs. In the first assay, the following primers were used in PCR (conditions available upon request) to produce either a 380 or 390 bp length product: AGCCACTTTCGGGATTCTTC (F) and TGTTCCCTTGGTCACAGGAC (R). PCR products were then digested with 3 U *Apa*LI enzyme (NEB, Ipswich, MA, USA) at 37°C for three hours with standard NEB reaction conditions. Digested products were visualized on 2% agarose gel: fragments with the deletion are 354 bp in length and fragments without the deletion are 307 bp in length. In the second assay, fragment size analyses was performed for the genotyping of the *ARHGEF10* deletion (primers: CGGGTCTTCATGCTCAGTG (F) and TGTTCCCTTGGTCACAGGAC (R)) on an ABI 3730 capillary sequencer and analyzed with the GeneMapper 4.0 software (Life Technologies, Grand Island, NY, USA).

The deletion was further confirmed in cDNA prepared from tissue with RT-PCR. Total RNA was isolated under RNase-free conditions from flash frozen brain (Cavalier King Charles Spaniel, a control dog), spinal cord (Leonberger, an *ARHGEF10 D/N* dog with no polyneuropathy), and peripheral nerve tissue (Leonberger, one PN affected dog with *ARHGEF10 D/D* genotype) using Trizol's (Life Technologies, Grand Island, NY, USA) manufacturer instructions. Total RNA was reverse transcribed into cDNA using the Super Script II kit (Life Technologies, Grand Island, NY, USA), following the manufacturer's standard protocol. cDNA was then subjected to PCR and sequenced using intron-spanning primers (GCGGTCCGACGATATGATAG – F, ACACCTGCTTTCTCCAGCAC – R) located in exon 17 and 19.

Throughout the manuscript, all *ARHGEF10* cDNA numbering refers to accession XM_846774.3 and all protein numbering refers to accession XP_851867.3.

### Additional Genotyping of a CFA7 Locus

An RFLP assay specific to a SNP (TIGRP2P93473_rs9189862) tagging the originally significantly associated CFA7 haplotype was developed in order to genotype a larger population. The following primers were used in PCR (conditions available upon request) to produce a 290 bp product: AGATTCCCAATCCCTGCTTC (F) and AGGCAGGGCTATTCTTTTGG (R). PCR products were then digested with 5 U *Hae*III enzyme (NEB, Ipswich, MA, USA) at 37°C for three hours with standard NEB reaction conditions and visualized on 2% agarose gels.

### Web Resources

Inherited Peripheral Neuropathies Mutation Database (URL: http://www.molgen.ua.ac.be/cmtmutations/home/IPN.cfm)

University of California, Santa Cruz Genome Browser (URL: www.genome.ucsc.edu)

American Kennel Club Leonberger Breed History (URL: http://www.akc.org/breeds/leonberger/history.cfm)

Primer 3 (URL: http://frodo.wi.mit.edu/primer3/)

RepeatMasker (URL: http://repeatmasker.genome.washington.edu)

## Supporting Information

Supporting Information S1 LPN1 genetic test result interpretation and breeding recommendations.
(PDF)

Table S1 All observed Polymorphisms in *ARHGEF10* sequencing in case and control Leonbergers.
(XLSX)

Table S2 Number of dogs screened for ARHGEF10 deletion.
(XLSX)
