# Supplementary material for: An ARHGEF10 Deletion Is Highly Associated with a Juvenile-Onset Inherited Polyneuropathy in Leonberger and Saint Bernard Dogs
Source: PLoS Genet. 2014 Oct 2;10(10):e1004635. doi: 10.1371/journal.pgen.1004635 (PMC4183422; doi:10.1371/journal.pgen.1004635)
Supplement: Supporting Information S1 — LPN1 genetic test result interpretation and breeding recommendations. (PDF) [file pgen.1004635.s001.pdf]

LPN1 Genetic Test Result Interpretation

Clear (N/N): A clear dog has two copies of the normal gene (this is also referred to as being homozygous normal). However, this result does not rule out the possibility that a dog could have, or be a carrier for, a different polyneuropathy mutation that this test cannot detect.

Carrier / At Risk (D/N): A carrier dog has one copy of the normal form of the gene and one copy of the mutated form of the LPN1 gene (this is also referred to as being heterozygous). Our research population indicates approximately 25% of Leonbergers with a polyneuropathy diagnosed by nerve and muscle biopsy have one copy of the LPN1 gene mutation. However, whether the polyneuropathy in carrier dogs is due to this single copy of the LPN1 gene itself, or is a different form of neurological disease entirely, is not yet known. The average age that owners first notice clinical signs in carrier dogs, if they develop at all, is 6 years. Additionally, these dogs typically have milder clinical signs than dogs with two copies of the LPN1 mutation. Having one copy of the mutated form of the LPN1 gene does not rule out the possibility that a dog may have young onset of a different polyneuropathy caused by a mutation not detected by this test. LPN1 carriers will, on average, pass the LPN1 gene mutation on to half of their offspring.

Affected (D/D): An affected dog has two copies of the LPN1 gene mutation (this is also referred to as being homozygous affected). Affected dogs typically develop neurological disease at or before 3 years of age (average 1.5 years of age), and clinical signs tend to be severe, often requiring surgical intervention of laryngeal paralysis. Affected dogs will pass one copy of this mutation on to all of their offspring.

Further Information

We are testing for a specific DNA segment deletion in a specific gene; therefore this can be referred to as a gene mutation test. This situation is different from other types of genetic tests that describe only the identification of a DNA marker that could be very far away from the true disease gene, and not be as highly predictive as desired.

We have designated the letter D to indicate the mutant (LPN1) form of the gene and N to indicate the normal form of the gene. A dog's particular combination of N or D forms of the gene is known as its genotype. The genotype of a normal dog is designated as N/N and is clear of the mutation. N/N dogs do not have LPN1, however, some do develop neuropathy with similar clinical and histopathological signs due to other as-yet-unidentified mutations. All dogs with the D/D genotype identified to date are affected with LPN1 and have developed clinical signs of neurologic disease, typically by 3 years of age or younger. Dogs with the D/N genotype are currently considered carriers. At present, approximately 25% of dogs in our research population with polyneuropathy diagnosed by nerve and muscle biopsy have the D/N genotype. The average age that clinical signs are first noted in these D/N dogs is 6 years. D/N dogs typically present with less severe clinical signs than D/D dogs.

Due to other causes of neuropathy in Leonbergers, the exact mode of inheritance of the LPN1 form of neuropathy cannot yet be stated for certain. While it is possible that LPN1 is dominantly inherited, with a dose dependent nature to the disease (more copies = worse disease), current data more strongly supports that LPN1 is recessive, and that only D/D dogs have this form of neuropathy, while D/N and N/N dogs with clinical signs have another form of neuropathy. With either inheritance model, producing a puppy with severe, early-onset LPN caused by the mutant LPN1 gene would require that both parents be either carriers (D/N) or affected (D/D).

On the following page are the chances any given puppy in a litter from the indicated mating will have the genotype of clear, carrier or affected. Matings that produce, or are comprised of an affected dog are not recommended.

* Clear (N/N) x Clear (N/N) = 100% Clear (N/N)
* Clear (N/N) x Carrier (D/N) = 50% Clear (N/N), 50% Carrier (D/N)
  (This is an average, individual litters may see anywhere from 100% Clear to 100% Carrier)
* Clear (N/N) x Affected (D/D) = 100% Carrier (D/N)
* Carrier (D/N) x Carrier (D/N) = 25% Clear (N/N), 50% Carrier (D/N), 25% Affected (D/D)
  (This is an average, individual litters may see more or less of any result)
* Carrier (D/N) x Affected (D/D) = 50% Carrier (D/N), 50% Affected (D/D)
  (This is an average, individual litters may see anything from 100% Carrier to 100% Affected)
* Affected (D/D) x Affected (D/D) = 100% Affected (D/D)

Breeding Recommendations

Until other potential disease-causing mutations are discovered, it is impossible for us to determine with certainty why some D/N dogs develop disease. It could be due to the single copy of the LPN1 deletion, or it could be due to another form of disease (yet to be elucidated), or even a combination of the two.

As long as it is not absolutely clear that D/N dogs will all develop neurological disease, we recommend keeping them in the breeding pool for at least one generation. Litters of D/N x N/N matings should be tested and preferentially the N/N pups (50%) should be kept for future breeding. Immediately eliminating all D/N dogs from breeding may have negative consequences for the genetic diversity of the breed. Important lines within the breed should be maintained; for example, if an important line is about to vanish, limited use of D/N animals may be used to preserve them.

In other cases, it may be appropriate not to use any D/N dogs for breeding, if you "only" lose 15% of your population. This is not an overly dramatic loss of genetic diversity. Our recommendations were originally more cautious, because we did not know the exact frequency of carrier animals. As the predicted carrier rate has dropped from 25% to the more current estimate of 15%, this presents a good scenario for the sustainable eradication of LPN1.

We suggest a mid-term goal of reduction of the LPN1 allele from the breeding population. If eradication of LPN1 is desirous for a breed club, a complete ban of D/N animals starting in several years would represent a sensible measure in our opinion.

One final word of caution:

It is important to remember that this LPN1 test is diagnostic for only one of possibly several genetic risk factors for polyneuropathy. Thus, it is still possible that affected offspring with a different genetic form of polyneuropathy could occur, even from a mating of two dogs that both have been tested N/N for the LPN1 mutation. To that end, we also recommend that both dogs in a breeding pair be free of any signs of neurological disease, regardless of genotype, because this test can only detect one polyneuropathy mutation.

For additional information please refer to the following website:
www.vdl.umn.edu/vdl/ourservices/canineneuromuscular

This genetic test is performed in a research laboratory where procedures are not required to meet University of Minnesota Veterinary Diagnostic Laboratory regulatory requirements. However, the test method is conducted according to validated standard operating procedures and meets the research laboratory's quality control standards. We are available to answer any questions clients may have related to the testing procedure.
